# Differential Impact of Social and Monetary Reward on Procedural Learning and Consolidation in Aging and Its Structural Correlates

**DOI:** 10.3389/fnagi.2019.00188

**Published:** 2019-07-30

**Authors:** Christopher E. J. Doppler, Linda Meyer, Anna Dovern, Jaro Stühmer-Beckh, Peter H. Weiss, Gereon R. Fink

**Affiliations:** ^1^Cognitive Neuroscience, Institute of Neuroscience and Medicine (INM-3), Jülich Research Centre, Jülich, Germany; ^2^Department of Neurology, Faculty of Medicine, University Hospital Cologne, University of Cologne, Cologne, Germany; ^3^Department of Neurology, Klinikum Leverkusen, Leverkusen, Germany; ^4^Department of Ophthalmology, University Hospital Würzburg, Würzburg, Germany

**Keywords:** serial reaction time task, procedural learning, reinforcement learning, voxel-based morphometry, motor aging

## Abstract

In young (*n* = 36, mean ± SD: 24.8 ± 4.5 years) and older (*n* = 34, mean ± SD: 65.1 ± 6.5 years) healthy participants, we employed a modified version of the Serial Reaction Time task to measure procedural learning (PL) and consolidation while providing monetary and social reward. Using voxel-based morphometry (VBM), we additionally determined the structural correlates of reward-related motor performance (RMP) and PL. Monetary reward had a beneficial effect on PL in the older subjects only. In contrast, social reward significantly enhanced PL in the older and consolidation in the young participants. VBM analyses revealed that motor performance related to monetary reward was associated with larger grey matter volume (GMV) of the left striatum in the young, and motor performance related to social reward with larger GMV of the medial orbitofrontal cortex in the older group. The differential effects of social reward in young (improved consolidation) and both social and monetary rewards in older (enhanced PL) healthy subjects point to the potential of rewards for interventions targeting aging-associated motor decline or stroke-induced motor deficits.

## Introduction

The preservation of acquired as well as the acquisition of new motor skills are essential across the entire lifespan. In particular, when aging-associated impairments of motor function occur or when aging-associated diseases, e.g., a stroke, impact upon the motor system, training of previously learnt motor skills or the acquisition of new motor skills become necessary. Given the sociodemographic changes and the resulting increasing incidence of stroke (Struijs et al., 2005), a deeper understanding of the processes affecting procedural learning and the effects of aging thereon is of paramount importance. Moreover, the ongoing debate about the effectiveness of different concepts of physical rehabilitation (Pollock et al., 2014) and the limited evidence for the effectiveness of emerging new interventions (Chang and Kim, 2013) call for further improvements in procedural learning in rehabilitation.

One way to enhance procedural learning and consolidation, the process leading to the retention of the learnt motor knowledge (Robertson et al., 2004; Robertson, 2009), is the application of reward. During the last decade, the influence of reward on procedural and motor learning as well as on consolidation has been studied intensively (O’Doherty et al., 2001; Wickens et al., 2003; O’Doherty, 2004; Abe et al., 2011; Palminteri et al., 2011; Nikooyan and Ahmed, 2015). Many of these studies used monetary reward (Wächter et al., 2009; Abe et al., 2011; Palminteri et al., 2011). In the clinical setting, however, an important aspect distinguishing conventional physical rehabilitation from, for instance, robot-assisted therapies is the rewarding nature of social interactions with the therapists. Yet, the literature about the effect of social reward on procedural learning remains scarce. To our knowledge, there is only one study with young, healthy subjects showing an enhancement of motor memory consolidation by social reward (Sugawara et al., 2012). For the effects of monetary reward on procedural learning and consolidation more evidence can be found – albeit with diverging results. Monetary reward improved procedural learning (Wächter et al., 2009) and consolidation (Abe et al., 2011) in young subjects. However, another study showed a beneficial effect of monetary reward on procedural learning, but not on consolidation in a sample of young participants (Steel et al., 2016).

Unfortunately, most studies investigated young participants only. A recent study has shown that the application of monetary reward can support procedural learning and retention in patients with stroke (Quattrocchi et al., 2017). Concerning the differential effects of social and monetary rewards in aging, a recent fMRI study adopting an incentive delay task offering different rewards demonstrated an interaction of reward type and age on the neural activity of the Nucleus accumbens (NAcc). In the older subjects, social reward cues led to enhanced NAcc activation, while monetary reward cues increased NAcc activation in young subjects (Rademacher et al., 2014). Besides these data about the effects of reward on behavioural tasks, the socioemotional selectivity theory represents a theoretical background for differential susceptibility to rewards depending on age and its effect on motivation (Carstensen, 2006), stating that during aging priorities shift to receiving readily available rewards.

Taken together, these previous findings suggest age-dependent effects of social and monetary reward on procedural learning and consolidation. Accordingly, we compared these two kinds of rewards in younger and older subjects. Putative differential, age-related effects of social and monetary rewards on procedural learning and consolidation might eventually be used to counteract aging- or disease-associated impairments of motor function.

To investigate the specific effects of reward on procedural learning and consolidation in aging, we used a modified version of the serial reaction time (SRT) task (Nissen and Bullemer, 1987). According to previous studies (Robertson, 2007; Dovern et al., 2011), we operationalized procedural learning by comparing the reaction times for blocks with a repeating sequence to those with random trials. Thus, this operationalized procedural parameter represents sequence-specific learning in the SRT task. The SRT task was combined with the performance-dependent application of monetary and social rewards as well as neutral feedback in younger and older participants and was performed on two consecutive days (day 1: assessment of procedural learning, day 2: assessment of consolidation). According to previous studies, we hypothesised a beneficial effect of these rewards on sequence-specific learning on day 1 (representing procedural learning) and on the retrieval of sequence-specific knowledge on day 2 (representing consolidation). Besides the effects of monetary and social reward on procedural learning and consolidation, we examined the individual reward-related motor performance.

Anatomical studies suggest an association between regional brain volumes and the performance in motor tasks (Kennedy and Raz, 2005; Raz and Rodrigue, 2006) as well as the sensitivity to reward (Parvaz et al., 2012). This association is supported by a decrease of grey matter volume (GMV) in people with cocaine abuse, who have impaired reward processing (Parvaz et al., 2012). Also, Lebreton et al. (2009) described a structural disposition of the reward system to social interaction. Therefore, we hypothesised an association between the GMV in key areas of the reward system and (i) motor performance related to social and monetary rewards, and (ii) procedural learning under social and monetary reward.

## Materials and Methods

### Participants

Seventy-two healthy participants took part in the study. The study population consisted of two groups: one with young (*n* = 36, 18 men; age (mean ± SD): 24.8 ± 4.5 years, range: 18–35 years) and one with older participants (*n* = 36). Exclusion criteria were any history of a neurological or psychiatric disorder or the use of psychopharmacologically active medication. In the group of older subjects, one subject had to be excluded from further analysis due to technical issues, another one due to an error rate >3 standard deviations from the mean of the older group. Thus, data from 34 older subjects entered the subsequent analyses (21 men; 65.1 ± 6.5 years, range: 54–81 years). Structural MRI scans were acquired for a subset of 28 young and 32 older participants. Using Oldfield’s laterality quotient (Oldfield, 1971), we identified participants as left-handed (<−50), right-handed (>50), or ambidextrous (−50 to 50) as proposed by Dragovic (2004). According to this classification, 4 (5.7%) subjects were left-handed. This distribution is in line with the distribution of left-handedness in samples of this age (Fleminger et al., 1977). All subjects performed the task with their dominant hand.

The group of older participants underwent a dementia screening (Mini-mental state examination, MMSE) and all (young and older) participants filled out the Beck Depression Inventory (BDI) to rule out clinically relevant symptoms of depression as well as the German version of the Barratt Impulsiveness Scale (BIS-11) (Preuss et al., 2008). Each participant also performed the Corsi block-tapping test to assess her/his visuo-spatial short-term working memory (Corsi, 1973).

All subjects gave written informed consent before their participation. The ethics committee of the Faculty of Medicine, University of Cologne, had approved the study.

### Behavioural Task

We used a modified version of the Serial Reaction Time (SRT) Task (Nissen and Bullemer, 1987). Subjects saw stimuli presented at one of three horizontally arranged target positions presented on the 14′′ TFT screen of a standard notebook (viewing distance: 70 cm) running Presentation^®^ (Version 14.9, Neurobehavioral Systems). Subjects were instructed to press the spatially congruent button on a custom-made three-button-keyboard as fast as possible. When the response in the trial was correct and the reaction time (RT) was lower than the individual median RT (as established in a practice session with 45 trials taking place on day 1 before the start of the experiment), a feedback was shown for 250 ms followed by an inter-stimulus interval of 250 ms. The median RT of the practice session was used to reduce the impact of potential outliers (especially extremely long RTs) on the criterion that triggered the application of reward/feedback in the experimental blocks.

**FIGURE 1:**
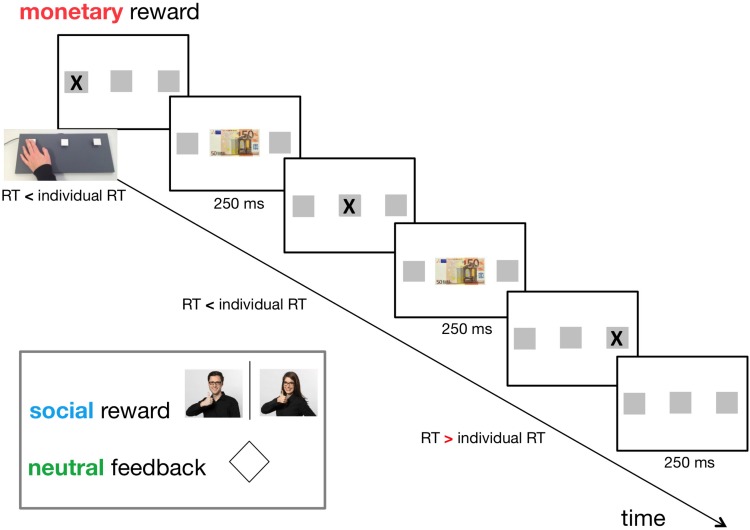
Timeline of the adapted version of the Serial Reaction Time Task (SRT) (day 1). Individual reaction time (RT) had been established in a practice session consisting of 45 random trials before the actual experiment. Three types of feedback were applied in a within-subject design: neutral feedback (for control), social reward, and monetary reward. The feedback was displayed for 250 ms if the participant pressed the correct button on a custom-made three-button keyboard faster than her/his individual RT. An interstimulus interval of 250 ms followed this. In the monetary reward condition, a 50 € banknote was displayed at the centre of the screen. In the social reward condition, a photo of one investigator (CEJD, LM) giving positive feedback was shown. For neutral feedback, a rhombus was displayed at the centre of the screen.

We used three types of feedback: a monetary reward, a social reward, and a neutral reward/feedback (for control). In the monetary reward condition, a 50 € banknote was displayed at the centre of the screen. Participants were informed that every time they saw the 50 € note, their pay would increase by 0.05 €. In the social reward condition, a photo of one investigator (CEJD, LM) giving positive feedback was shown (Figure 1). The use of the investigators’ photographs was counterbalanced across female/male participants. For the neutral reward, a rhombus was displayed at the centre of the screen (Figure 1).

Stimuli were presented either in a repetitive sequential manner (S) or a pseudo-random (R) succession, but subjects were not informed about the presence of a sequential pattern. Three different 9-element sequences were used; one for each reward/feedback condition (counterbalanced across subjects). Each reward/feedback condition consisted of seven blocks: R1-S2-S3-S4-s5-r6-s7. During the first four blocks (R1–S4) reward/feedback was presented to promote reward-based procedural learning. The last three blocks (small letters assigned: s5, r6, and s7) were used to assess the sequence-specific learning effect. To avoid a confound of reward/feedback on the retrieval of the learnt sequences, we chose not to apply reward/feedback during these three blocks. We added block s7 to rule out that fatigue effects provoked a potential increase in RT between the blocks used for calculation of the sequence-specific learning effect (s5, r6). Within the five sequence blocks (S2, S3, S4, s5, s7), each 9-element sequence was repeated five times per block, which resulted in twenty-five repetitions of each sequence in total. Breaks separated the blocks. The subjects could freely choose the length of the break by pressing the enter key to continue with the next block. The order of reward/feedback conditions was counterbalanced across subjects. This means that the three reward/feedback conditions were performed in separate sessions, each consisting of seven blocks (i.e., the five sequence blocks and the two random blocks). Every possible combination of reward/feedback conditions (six combinations: monetary-neutral-social, monetary-social-neutral, neutral-monetary-social, neutral-social-monetary, social-monetary-neutral, social-neutral-monetary) and sequences (also six combinations: 123, 132, 213, 231, 312, 321) was equally distributed across subjects. Each given combination was performed by six subjects, except the combinations where participants had to be excluded as mentioned above.

On day 2, subjects performed a shortened version of the same task (r1-s2-r3-s4-r5-s6-r7, again counterbalanced between subjects) and without any feedback, using the same three sequences as on day 1 (i.e., the sequences learnt in the three reward/feedback conditions on day 1) in the same order as on day 1 to investigate consolidation. In contrast to day 1 where multiple “sequence blocks” were repeated, we chose this alternating structure of blocks to avoid further sequence-specific learning on day 2 but rather concentrated on assessing consolidation. We used multiple “random blocks” to rule out RT effects caused by fatigue or general practice.

We used three different 9-element sequences: 2-1-3-3-2-2-3-1-1, 3-2-3-3-1-2-2-1-1, and 3-3-1-3-2-2-1-1-2 (“1” represents the left location of the stimulus on the screen, “2” the middle location, and “3” the right location). All sequences – including the ones used in the r/R blocks – were constructed according to the following rules as applied in previous studies (Dovern et al., 2011) to avoid possible confounds by differences in the structure of the sequences:

(1) No more than two repeats of one stimulus location (e.g., …1-1-1… not allowed).
(2) No more than two unidirectional stimulus locations in a row (e.g., 1-2-3… not allowed).
(3) No repeated doublets (e.g., 1-2-1-2… not allowed).
(4) No three repetitions in a row (e.g., 2-2-1-1-3-3… not allowed).
(5) Each stimulus location had to appear three times in every sequence.
(6) Each stimulus location occurred three times, each time followed by a different stimulus location.

In other words, the frequency of all possible stimulus locations and the transition probabilities between consecutive stimulus locations were equally distributed across sequences.

After completion of the task, explicit motor sequence knowledge was assessed on day 2 using a standardised structured interview and a free recall test. First, we asked the participants whether they had recognised something about the experiment that we had not told them. Secondly, if the participants did not report any irregularities, we asked the participants whether they had recognised something about the succession of the stimuli. If participants stated that they had not recognised anything about the succession of the stimuli, we asked the participants whether they thought that the succession had been random or not. Participants who still described the succession as being random were told about the underlying structure of the experiment. All participants were then asked to reproduce at least one of the sequences used (by ticking off the positions of the stimuli on a sheet of paper) and to assign the associated reward condition. Participants were allowed to reproduce as many of the three sequences as they wished. We operationalized explicit motor sequence knowledge as the highest count of correct items in a row.

### Statistical Analysis

SPSS^®^ 21 (IBM) and RStudio 0.98 were used to analyse the behavioural data. The first trial of each block was rejected. Furthermore, error trials and the respective following trial were removed, and the median RT per block was calculated for every subject. Again, the median RT per block was used to reduce the impact of potential outliers (especially extremely long RT). These were then used to calculate the mean RT across subjects for every block. Similar to previous studies investigating procedural learning (Dovern et al., 2011; Meier and Cock, 2014), we examined the general learning and sequence-specific learning effects. General learning (GL) indicates the effect of habituation to the task. Sequence-specific learning (SSL), however, represents reaction time gains due to specific knowledge about the sequences learnt by the participants. On day 1, the GL effect was calculated by subtracting the median RT of block S4 from the median RT of block S2. The SSL effect (indicating procedural learning) was represented by the difference of the median RT of block r6 (pseudo-random succession) and the mean of the median RTs of its preceding (s5) and succeeding (s7) sequence blocks. We used block s7 for this calculation to avoid a potential confound by an increase of RT in block r6 due to fatigue. On day 2, the median RT of the respective sequence block was subtracted from the mean of the median RTs of blocks r1, r3, r5, and r7 to calculate the SSL effect (indicating consolidation). To assess the impact of the different feedback/rewards on learning, we performed repeated-measures ANOVAs for both groups separately (within-subject factor reward with three levels: neutral, monetary, and social reward). We used Bonferroni correction for the *post hoc t-*tests between the three reward conditions.

We operationalized motor performance related to monetary and social rewards as the percentage of rewarded trials of each participant in the monetary and the social reward conditions, respectively. A correlation analysis between reward-related motor performance (RMP) and general as well as sequence-specific learning for both groups revealed no significant correlations (all *p* > 0.1) suggesting that RMP represents not a mere improvement in task performance or motor sequence knowledge. As there is evidence for an association between reward sensitivity and impulsiveness in an event-related potentials (ERP) study (Martin and Potts, 2004), we furthermore examined the association between impulsiveness, according to the Barratt Impulsiveness Scale (BIS-11), and RMP. Specifically in older participants, motor performance related to social reward correlated with impulsiveness (*r* = 0.423; *p* < 0.05).

### Voxel-Based Morphometry

#### Image Acquisition

Magnetic resonance imaging was conducted on a 3.0 T Trio scanner (Siemens, Erlangen, Germany) using a 12-channel Siemens head coil. The structural T1-weighted magnetisation prepared gradient-echo images were acquired using the following parameters: repetition time (TR) 2250 ms; echo time (TE) 3.03 ms; field of view (FOV) 256 mm; 176 sagittal slices of 1 mm thickness; flip angle = 9°, voxel size 1 × 1 × 1 mm. We screened MR images visually for artefacts and to exclude pathology.

#### Image Preprocessing

Data were processed and analysed using the Statistical Parametric Mapping software (SPM8; Wellcome Department of Imaging Neuroscience, London, United Kingdom)^1^. Subsequently, the VBM8 toolbox (VBM; Structural Brain Mapping Group, Jena, Germany)^2^ was employed using the default parameters. Images were first reoriented and aligned to the anterior commissure, followed by segmentation into grey matter (GM), white matter (WM), and cerebrospinal fluid (CSF). The resulting images were normalised to the stereotactic space of the Montreal Neurological Institute (MNI) using the iterative high-dimensional “Diffeomorphic Anatomical Registration Through Exponentiated Lie Algebra” (DARTEL) normalisation procedure. As spatial normalisation expands and contracts some brain regions, modulation was performed subsequently. This involves scaling by the amount of concentration so that the total amount of GM in the modulated GM remains the same as it was in the original images. Checking sample homogeneity resulted in the exclusion of one older participant. Modulated GM segments of 30 older and 28 younger participants were then smoothed using a Gaussian kernel with a value of 10-mm full-width as recommended by the VBM8-Toolbox Manual.

#### Statistical Analysis of Voxel-Based Morphometry

Voxel-based morphometry (VBM) was used to examine the neural correlate of RMP and the susceptibility to a given type of feedback during procedural learning at different ages. We, therefore, assessed the relation of these parameters with GMV for both age groups separately. The behavioural data of young and older participants were split into terciles depending on the percentage of rewarded trials (RMP) or the SSL effect (procedural learning) the participants achieved in each reward condition on day 1. Separately for young and older participants, we then compared the GMV of participants in the first tercile (with low RMP, i.e., a low number of rewarded trials) with the GMV of the respective participants in the third tercile (with high RMP, i.e., a high number of rewarded trials). The same was also done for the SSL effects on day 1. Using SPM8, we conducted a two-sample *t*-test with the smoothed GM images to investigate the association of GMV with the participants’ behavioural measures.

#### Region of Interest Analysis

Based on previous studies (O’Doherty, 2004; Izuma et al., 2008; Lebreton et al., 2009; Rademacher et al., 2014), we performed a region of interest (ROI) analysis with the following key regions of the reward system (Haber and Knutson, 2009): medial orbitofrontal cortex (mOFC), striatum, amygdala, and nucleus accumbens (NAcc). ROI analyses were performed with the following masks: The ROI for the mOFC (MNI coordinates *x*, *y*, *z* = −6, 36, −15) was defined according to functional imaging data by Lin and colleagues (Lin et al., 2012). The ROIs for the NAcc (left NAcc: MNI coordinates *x*, *y*, *z* = −9, 6, −4, right NAcc: *x*, *y*, *z* = 9, 6, −4) were defined according to a stereotactic investigation (Neto et al., 2008). The WFU PickAtlas was employed to create the mOFC and NAcc ROIs using a 10 mm sphere around the respective coordinates. The left striatum mask and the bilateral amygdala mask were generated using MRIcron (Version 6) and the SPM anatomy toolbox (Eickhoff et al., 2005), respectively (for an overview of all masks see Supplementary Figure S1). The threshold of statistical significance was set to *p*_SVC_ < 0.05 (i.e., FWE-corrected for small volume).

## Results

### Neuropsychological Assessment

The two groups showed a significantly different performance in the Corsi block-tapping test [young subjects: 6.42 ± 1.08 (mean ± SD); older subjects: 4.97 ± 0.74; *t*(66) = 6.373; *p* < 0.001]. Neither group showed clinically relevant symptoms of depression in the BDI (cut-off > 9) (young subjects: 2.24 ± 2.12; older subjects: 3.63 ± 3.05). Moreover, the groups did not differ concerning impulsiveness, according to the Barratt Impulsiveness Scale (*t*(64) = 0.274; *p* = 0.785). All older subjects achieved unremarkable scores in the dementia screening test MMSE.

### Behavioural Data

All descriptive statistics are provided as mean ± standard error of the mean (SEM) unless stated otherwise.

#### Error Rates and Mean Reaction Times

On both days, there was no significant difference in error rates between groups (day 1: *t*(68) = 1.345; *p* = 0.183; day 2: *t*(68) = 1.286; *p* = 0.203). Moreover, the mean error rates across conditions were <1% for both groups (young: 0.64 ± 0.07%, older: 0.93 ± 0.16%). Due to this fact, we decided to restrict further analyses to reaction time (RT) data.

**FIGURE 2:**
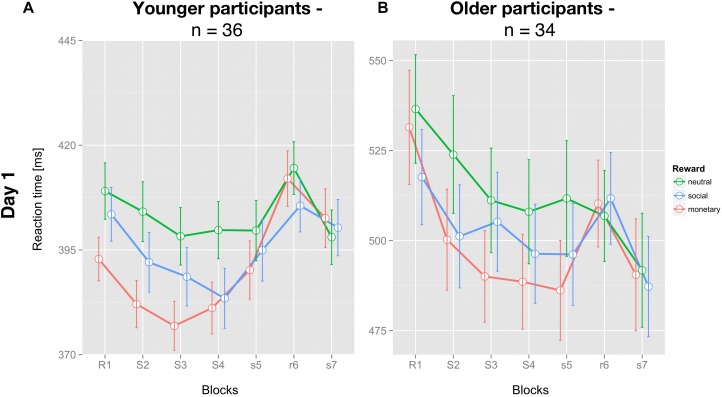
Average reaction times of the SRT paradigm for each reward condition and both groups on day one. **(A)** Mean reaction time of young (*n* = 36) and **(B)** older (*n* = 34) participants for each condition and block (± SEM) are depicted for all three feedback conditions: neutral feedback (green), social reward (blue), and monetary reward (red). Capital letters identify blocks with feedback, small letters blocks without feedback. S/s identifies blocks with a repetitive sequential pattern, R/r blocks with pseudo-random succession.

As expected, young participants had significantly faster mean RT (396.6 ± 5.9 ms; Figure 2A) than older participants (504.9 ± 13.1 ms; Figure 2B; *t*(68) = 7.670; *p* < 0.001).

#### General Learning/Sequence-Specific Learning

On day 1, irrespective of reward type, younger participants showed a significant GL (4.7 ± 2.2 ms; *t*(35) = 2.167; *p* < 0.05) and SSL (13.0 ± 3.0 ms; *t*(35) = 4.443; *p* < 0.001) effect. On day 2, the significant SSL effect had been retained in the young group (14.5 ± 3.8 ms; *t*(35) = 3.814; *p* < 0.01). The same applied to the group of older participants (GL on day 1: 10.8 ± 3.8 ms; *t*(33) = 2.815; *p* < 0.01; SSL on day 1: 15.7 ± 5.3 ms; *t*(33) = 2.990; *p* < 0.01; SSL on day 2: 10.3 ± 3.5 ms; *t*(33) = 2.968; *p* < 0.01).

Concerning GL on day 1, neither group showed a differential effect of reward (young: *F*(2,70) = 1.449; *p* = 0.242; older: *F*(2,66) = 1.339; *p* = 0.269).

**FIGURE 3:**
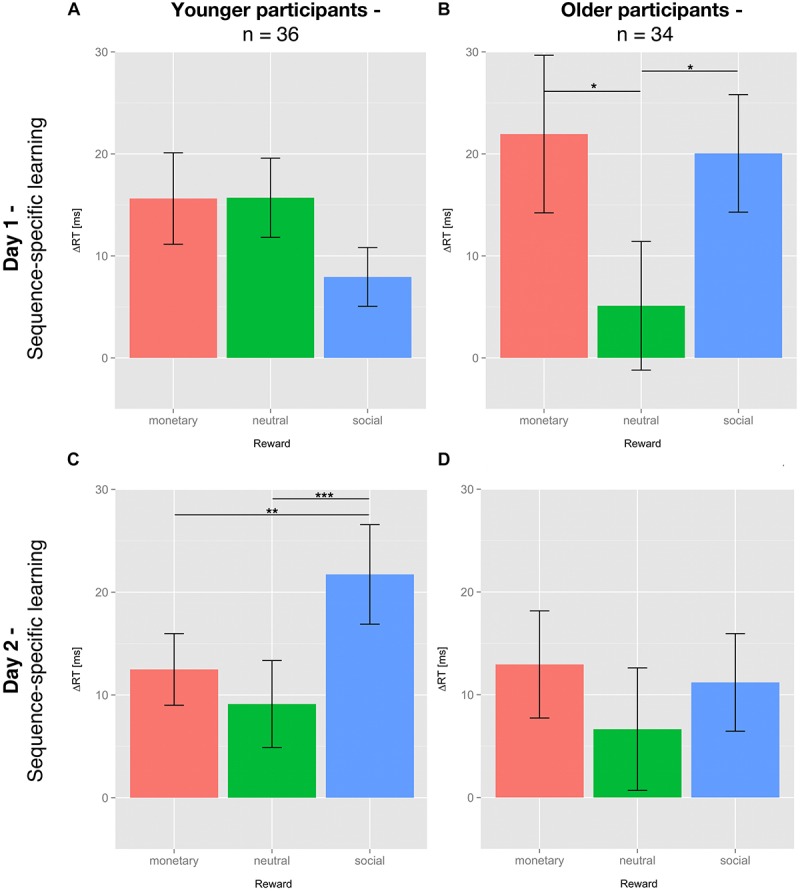
Sequence-specific learning effects of young and older subjects on day 1 and 2. Sequence-specific learning (SSL) effects are calculated by subtracting the mean of the median reaction time (RT) of sequence blocks s5 and s7 from the median RT of the random block r6 (day 1) or subtracting the median RT of the respective sequence block from the mean of the median RT of all random blocks (day 2). SSL effects are depicted for day 1 **(A,B)** and day 2 **(C,D)**, young **(A,C)** and older **(B,D)** participants and all three feedback conditions: neutral feedback (green), social reward (blue), and monetary reward (red). Displayed are the means ± SEM. The asterisks denote significant differences between rewards: ^*^p < 0.05; ^∗∗^*p* < 0.01; ^∗∗∗^*p* < 0.001.

For SSL on day 1, no significant differential influence of reward type (*F*(2,70) = 2.284; *p* = 0.109) was detected for the young group (Figure 3A). In contrast, for the older group there was a significant influence of reward type on SSL on day 1 (*F*(2,66) = 3.409; *p* < 0.05). *Post hoc* tests revealed a significantly higher SSL effect for social compared to neutral reward (*t*(33) = 2.554; *p* < 0.05, one-sided, Bonferroni-corrected) and for monetary vs. neutral reward (*t*(33) = 2.242; *p* < 0.05, one-sided, Bonferroni-corrected). SSL effects for monetary and social rewards on day 1 did not differ significantly in the older group (*t*(33) = 0.247, *p* = 0.8; Figure 3B).

We performed repeated-measures ANOVAs for general learning and sequence-specific learning on day 1 with the within-subject factor order of sequences/rewards and the between-subject factor group to assess potential order effects on the efficiency of the reward conditions. Neither for general learning there was a significant interaction (*F*(2,136) = 0.975, *p* = 0.380) or a significant main effect of order (*F*(2,136) = 0.105, *p* = 0.900), nor for sequence-specific learning (interaction: *F*(2,136) = 1.333, *p* = 0.267; main effect of order: *F*(2,136) = 0.170, *p* = 0.844).

#### Reward-Related Motor Performance

To analyse the RMP of the participants, we calculated the rate of rewarded trials separately for the three reward conditions on day 1. Repeated-measures ANOVAs with the within-subject factor reward (neutral, monetary, or social reward) and the between-subject factor group revealed significant main effects of reward (*F*(2,136) = 14.703; *p* < 0.001) and group (*F*(1,68) = 15.803; *p* < 0.001). The latter resulted from a significantly higher proportion of rewarded trials in young compared to older participants. A comparison of the three reward types using *post hoc t-*tests revealed that the young group showed the highest proportion of rewarded trials in the monetary reward condition (81.0 ± 1.8%; vs. neutral feedback: 71.4 ± 2.5%; *t*(35) = 5.760; *p* < 0.001, Bonferroni-corrected; vs. social reward: 76.4 ± 2.3%; *t*(35) = 2.495; n. s.), followed by the social reward condition (vs. neutral feedback: *t*(35) = 2.625; *p* < 0.05, Bonferroni-corrected). The older group presented the same tendency, however, only the proportion of rewarded trials in the monetary reward condition was significantly higher than the one in the neutral feedback condition (monetary: 66.0 ± 2.7%; neutral: 60.1 ± 3.0%; social: 63.4 ± 3.1%; monetary vs. neutral: *t*(33) = 2.575; *p* < 0.05, Bonferroni-corrected).

#### Effects of Age and Reward on Sequence-Specific Learning and Consolidation

The effects of the different types of reward on consolidation, operationalized by SSL on day 2, did no longer reach significance in the older participants (*F*(2,66) = 0.428; *p* = 0.654; Figure 3D). In the young group, however, the application of reward during the learning phase on day 1 had a differential impact on consolidation on day 2 (*F*(2,70) = 8.122; *p* = 0.001; Figure 3C). One-sided *post hoc t*-tests revealed that consolidation of the motor sequence knowledge learnt under social reward was significantly higher than that associated with neutral feedback (*t*(35) = 3.651; *p* < 0.01) or monetary reward (*t*(35) = 2.879; *p* < 0.01). Consolidation of motor sequence knowledge learnt under monetary reward did not differ from consolidation associated with neutral feedback (*t*(35) = 1.105; *p* = 0.139, all Bonferroni-corrected).

To assess potential changes in SSL from day 1 to day 2 and the effects of age and reward thereon, we performed a mixed-design ANOVA with the between-subjects factor group (older vs. young) and the within-subjects factors day (day 1 vs. day 2) and reward (neutral, monetary, or social). There was a significant three-way interaction day × reward × group (*F*(2,136) = 4.717; *p* = 0.010). This interaction was driven by the fact that only in the young group, there was a specific enhancement of the SSL effect from day 1 to day 2 for the sequences learnt under social reward (*t*(35) = 3.270; *p* < 0.01, Bonferroni-corrected). The main effects of group (*F*(1,68) = 0.025; *p* = 0.874), day (*F*(1,68) = 0.625; *p* = 0.432) and reward (*F*(2,68) = 2.959; *p* = 0.55) as well as all two-way interactions were not significant (*p* > 0.05).

To elucidate potential causes for the lack of differential effects of reward on consolidation in the older participants, we analysed SSL on day 2 with a different approach. To this end, we used the order of sequences on day 1 (irrespective of the type of reward associated with a given sequence) as the independent variable. Especially in the older group, consolidation critically depended on the order of sequences on day 1 (*F*(2,66) = 6.279; *p* < 0.01), as SSL for the first sequence learnt was significantly larger than SSL for the third sequence (one-sided *post hoc t*-test: *t*(33) = 2.914; *p* < 0.01, Bonferroni-corrected, see Supplementary Figure S2). In contrast, no main effect of sequence order was present in the young group (F(2,70) = 1.744; *p* = 0.182).

#### Explicit Motor Sequence Knowledge

The groups did not differ regarding the maximum number of correctly reproduced elements of the sequence in a row (*t*(66) = 0.100; *p* = 0.921), with a mean of 4.5 elements in each group (young group: SD 1.3, older group: SD 1.2). To assess a potential association between explicit motor sequence knowledge and SSL (on day 1), we performed a correlation analysis between the individual maximum number of correctly reproduced elements of the sequence in a row and the individual SSL. Only for the SSL in the neutral feedback condition, there was a significant, but negative correlation in the older group (*r* = −0.353; *p* < 0.05) indicating that the SSL of older subjects in the neutral feedback condition was lower when they achieved more explicit motor sequence knowledge. All other associations were not significant. Despite the notion that explicit motor sequence knowledge is an essential factor for motor/procedural learning (Haider and Frensch, 2009; Yordanova et al., 2015), the data suggest that there was no relevant association between explicit motor sequence knowledge and SSL on day 1 in the current study.

### Voxel-Based Morphometry

**FIGURE 4:**
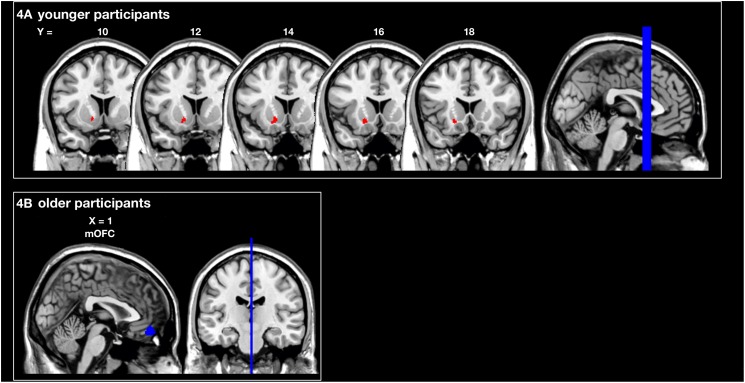
Region of interest (ROI) analysis of grey matter volume (GMV) differences between subjects grouped according to their reward-related motor performance (operationalized as the percentage of rewarded trials; third vs. first tercile). **(A)** Motor performance related to monetary reward (red) in young participants: left striatum (putamen). **(B)** Motor performance related to social reward (blue) in older participants: medial orbitofrontal cortex (mOFC). Displayed voxels survive FWE-correction within the ROIs (all *p*_SVC_ < 0.05). All images are displayed on coronal **(A)** and sagittal **(B)** slices of the standard brain provided by MRIcron in neurological convention. *x*- and *y*-coordinates refer to the MNI-space (SVC, small volume corrected).

The analysis of the structural correlates of motor performance related to social and monetary reward revealed that better motor performance related to monetary reward was associated with more GMV in the left putamen of young subjects (*p*_SVC_ < 0.05). In contrast, better motor performance related to social reward correlated with a larger GMV in the mOFC of older subjects (*p*_SVC_ < 0.05; all small volume-corrected with FWE-correction; see Figure 4 and Table 1). For the younger group, this corresponded behaviourally to a difference in rewarded trials under the monetary reward of 70.2 ± 2.5 vs. to 93.3 ± 1.1%. For the older group, the difference in rewarded trials under the social reward was 43.4 ± 2.1 vs. 81.8 ± 1.6%.

**TABLE 1:** Brain regions showing larger grey matter volumes associated with motor performance related to monetary/social reward and procedural learning.

**Brain region**	**Hemisphere**	**MNI coordinates**				
		***x***	***y***	***z***	**Peak *z*-score**	Cluster size^*^
**Motor performance related to monetary reward – younger participants**						
Striatum	Left	−18	17	−9	3.48	40
**Motor performance related to social reward – older participants**						
mOFC		0	44	−17	3.14	1
**Sequence-specific learning on day 1– monetary reward – younger participants**						
Striatum	Left	−26	15	−6	3.61	23
		−26	3	−12	3.56	22
		−6	8	−8	3.47	13
NAcc	Left	−2	6	−5	4.01	99
	Right	3	6	−5	3.79	15
Amygdala	Left	−26	0	−14	3.59	62
	Right	29	0	−12	4.33	82

The coordinates in MNI space refer to the voxel with the highest grey matter volume within the regions of interest [*x* = distance (mm) to right (+) or left (−) of the midsagittal plane; *y* = distance anterior (+) or posterior (−) to vertical plane through the anterior commissure; *z* = distance above (+) or below (−) the intercommissural (AC–PC) plane; mOFC, medial orbitofrontal cortex; NAcc, nucleus accumbens]. ^*^Note that this refers to the size of the overlap between ROI mask and VBM cluster.

Regarding procedural learning in young subjects, an association between a better SSL effect on day 1 under monetary reward and a larger GMV in the left striatum (*p*_SVC_ < 0.05) as well as Ncl. accumbens and amygdala bilaterally (*p*_SVC_ < 0.05; all small volume-corrected with FWE-correction; Table 1) was observed. For the younger group, this corresponded behaviourally to a difference in SSL under the monetary reward of −9.9 ± 16.8 vs. to 33.9 ± 12 ms.

All other VBM analyses revealed no significant results.

## Discussion

Our study is – to the best of our knowledge – the first to assess the impact of social and monetary reward on the learning and consolidation of motor sequence knowledge in two different age groups. Moreover, the current VBM analyses revealed structural differences in critical regions of the reward system associated with motor performance related to social reward in the older participants as well as with motor performance related to monetary reward and procedural learning in young subjects.

Consistent with previous studies examining age effects on SRT task performance that found similar procedural learning capabilities in younger and older subjects (Howard and Howard, 1989, 1992), younger and older subjects showed significant motor sequence learning on day 1 and consolidation of learnt motor sequence knowledge on day 2, irrespective of reward type in the current study. In the older group, social and monetary reward (compared to neutral feedback) enhanced procedural learning on day 1. In contrast, no differential impact of the applied rewards (relative to neutral feedback) on procedural learning was detected in the young group on day 1. The socioemotional selectivity theory provides a possible explanation for the differential effect of both rewards on procedural learning in young and older participants. This theory describes a shift of motivational aspects in aging with a preference for goals that can be realised shortly, as the time available is perceived as limited (Carstensen, 2006). This effect might contribute to the result that the older participants were particularly susceptible to immediately available rewards, like receiving money or positive social feedback.

However, social reward led to better consolidation in the young subjects on day 2 (even surpassing monetary reward). Note that no reward/feedback was given during the retrieval session on day 2. These findings are consistent with data providing evidence for an enhancement of consolidation by social reward in healthy young subjects (Sugawara et al., 2012). Furthermore, the fact that social reward was the only condition where young subjects even improved from day 1 to day 2 adds further evidence to the relevance of social reward for consolidation in the young. Conversely, despite the older subject’s higher RMP for both reward types on day 1, the older participants showed no reward-related enhancement of consolidation. A possible explanation for this result might be derived from the effect of the order of sequences learnt on day 1 on consolidation on day 2 (section Effects of Age and Reward on Sequence-Specific Learning and Consolidation and Supplementary Figure S2). In contrast to the younger participants, consolidation critically depended on the sequence order in the older participants. Given that we applied the three reward conditions in a within-subject design, it seems that the older participants were particularly prone to between-sequence interference in the process of consolidating the motor sequence knowledge. Although the order of reward types was counterbalanced across subjects, this interference might have counteracted a differential effect of reward on consolidation. In contrast, in the younger subjects consolidation of motor sequence knowledge was not significantly influenced by the order of sequences learnt on day 1. Besides this factor, the number of trials might have sufficed to evoke differential reward effects only in young but not in older subjects, given that the connection between reward and the particular sequence had been established in only 135 trials on day 1.

Studies investigating the effects of aging on motor performance reported a specific deterioration of consolidation in older subjects (Brown et al., 2009; Nemeth and Janacsek, 2011). Neuroimaging research suggests that this deterioration results from deficient corticostriatal networks (King et al., 2013). In contrast, our data did not show a general impairment of consolidation with age, since the current older subjects exhibited – irrespective of reward type – a significant SSL on day 2 indicating proper consolidation of the learnt motor sequence knowledge. Given the previous findings, the most parsimonious explanation of our data is that reward – or in our case, even the application of neutral feedback – can be used to counteract this previously described impaired consolidation of motor sequence knowledge with age. Future studies are warranted to investigate whether this effect, for example, results from enhancing corticostriatal connectivity (Reynolds and Wickens, 2002).

VBM revealed that RMP was differentially associated with increased GMV in two main regions of the reward system: whereas motor performance related to monetary reward was associated with increased GMV in the striatum of the young, motor performance related to social reward correlated with increased GMV in the mOFC of older subjects. The lack of a significant correlation between RMP and SSL or GL measures indicates that this parameter does not merely reflect a general ability to improve performance. Thus, the current results add to previous evidence for an association between personality traits and brain structure (Kaasinen et al., 2005; Gardini et al., 2009; Schuerbeek et al., 2011; Bjørnebekk et al., 2012). Specifically, Lebreton and colleagues have already described an association between GMV in the orbitofrontal cortex and social reward dependence as assessed by Cloninger’s temperament and character inventory in young subjects aged 33–35 years (Lebreton et al., 2009). Our study extends these findings by revealing a similar structural disposition to social reward for older subjects (aged 54–81 years). The association between motor performance related to monetary reward and GMV in the striatum of the current young subjects is in line with fMRI studies showing enhanced activation in the striatum following monetary reward (Delgado et al., 2003, 2004; Zink et al., 2004). Besides, a study by Martin and Potts revealed an increased reward sensitivity in highly impulsive subjects (Martin and Potts, 2004). Notably, unlike in our study, their concept of reward sensitivity was operationalized by a specific component of event-related potentials and not by performance in a motor task. However, these findings are consistent with the significant correlation between impulsiveness and motor performance related to social reward in the current older subjects and additionally validates our operationalization of the parameter. These results further support the construct of reward sensitivity according to Gray’s biopsychological theory of personality, which postulates that the behavioural activation system (BAS) regulates reward sensitivity (Gray, 1987; Gray and McNaughton, 2003). Accordingly, studies revealed an association of the BAS scale and an activation of the striatum when receiving monetary reward (Simon et al., 2010) as well as an association between sensitivity to rewards (e.g., money and social standing) and activations in the striatum and medial prefrontal cortex (Linke et al., 2010).

The activity in the retention interval, the length of the interval, and the daytime of testing represent critical factors for consolidation (Cohen et al., 2005). In the current study, we controlled for the daytime of testing on both days of the experiment and advised participants to maintain their normal sleep-wake rhythm. We did not control for measures of sleep quality and other activity during the test-retest-interval. This may limit the interpretation of the current results regarding consolidation. Future studies are warranted that control for this issue.

In conclusion, our data revealed age-dependent differential effects of social reward on motor performance: whereas social (and monetary) reward especially improved motor sequence learning in older participants, the consolidation of motor sequence knowledge was improved by social reward in young subjects only. These differential effects of social reward (on consolidation) in young and of both social and monetary reward (on procedural learning) in older subjects point to the potential benefit of rewards for interventions counteracting aging- or disease-related motor deficits, an issue which warrants further investigation.

## Ethics Statement

This study was carried out in accordance with the recommendations of the ethics committee of the Faculty of Medicine, University of Cologne, with written informed consent from all subjects. All subjects gave written informed consent in accordance with the Declaration of Helsinki. The protocol was approved by the ethics committee of the Faculty of Medicine, University of Cologne.

## Author Contributions

CD and LM conceived and designed the study, acquired the data, analysed the data, and drafted the manuscript. AD analysed the data and critically revised the manuscript. JS-B acquired the data. PW and GF conceived and designed the study, analysed the data, and critically revised the manuscript.

## Conflict of Interest Statement

The authors declare that the research was conducted in the absence of any commercial or financial relationships that could be construed as a potential conflict of interest.

## Footnotes

1 http://www.fil.ion.ucl.ac.uk/spm

2 http://dbm.neuro.uni-jena.de/vbm.html

## Supplementary Material

FIGURE S1 Masks for the region of interest (ROI) analysis of voxel-based morphometry (VBM) data. **(A)** Amygdala [provided by the SPM anatomy toolbox (Eickhoff et al., 2005)]. **(B)** left Striatum [generated manually with MRIcron (Version 6)]. **(C)** medial orbitofrontal cortex (mOFC) [defined according to functional imaging data (Lin et al., 2012)]. **(D)** Nucleus accumbens (NAcc) [defined according to a stereotactic investigation (Neto et al., 2008)]. The masks are displayed on axial **(A–C)** and sagittal **(D)** slices of the standard brain provided by MRIcron. *x*- and *z*-coordinates refer to the MNI-space.

FIGURE S2 Sequence-specific learning (SSL) effects of both groups on day 2 depending on the order of sequences learnt on day 1. Sequence-specific learning (SSL) effects for day 2 are calculated by subtracting the median reaction time (RT) of the respective sequence block from the mean of the median reaction times (RT) of all random blocks (r1, r3, r5, r7). SSL effects on day 2 are depicted for young **(A)** and older **(B)** participants depending on the order in which the sequences had been learnt on day 1. Depicted are the means ± standard error of the mean (^∗∗^*p* < 0.01).

## References

[B1] Abe M. Schambra H. Wassermann E. M. Luckenbaugh D. Schweighofer N. Cohen L. G. (2011). Reward improves long-term retention of a motor memory through induction of offline memory gains. *Curr. Biol.* 21 557–562. 10.1016/j.cub.2011.02.030 21419628 PMC3075334

[B2] Bjørnebekk A. Westlye L. T. Fjell A. M. Grydeland H. Walhovd K. B. (2012). Social reward dependence and brain white matter microstructure. *Cereb. Cortex* 22 2672–2679. 10.1093/cercor/bhr345 22156472 PMC4705342

[B3] Brown R. M. Robertson E. M. Press D. Z. (2009). Sequence skill acquisition and off-line learning in normal aging. *Plos One* 4:e6683. 10.1371/journal.pone.0006683 19690610 PMC2723909

[B4] Carstensen L. L. (2006). The influence of a sense of time on human development. *Science* 312 1913–1915. 10.1126/science.1127488 16809530 PMC2790864

[B5] Chang W. Kim Y.-H. (2013). Robot-assisted therapy in stroke rehabilitation. *J. Stroke* 15 174–181. 10.5853/jos.2013.15.3.174 24396811 PMC3859002

[B6] Cohen D. A. Pascual-Leone A. Press D. Z. Robertson E. M. (2005). Off-line learning of motor skill memory: a double dissociation of goal and movement. *P. Natl. Acad. Sci. U. S. A.* 102 18237–18241. 10.1073/pnas.0506072102 16330773 PMC1312380

[B7] Corsi P. (1973). *Human Memory and the Medial Temporal Region of the Brain.* Ph.D. Theis, McGill University, Montreal, QC

[B8] Delgado M. Locke H. Stenger V. Fiez J. (2003). Dorsal striatum responses to reward and punishment: effects of valence and magnitude manipulations. *Cogn. Aff. Behav. Neurosci.* 3 27–38. 10.3758/cabn.3.1.27 12822596

[B9] Delgado M. R. Stenger V. A. Fiez J. A. (2004). Motivation-dependent responses in the human caudate nucleus. *Cereb. Cortex* 14 1022–1030. 10.1093/cercor/bhh062 15115748

[B10] Dovern A. Fink G. R. Saliger J. Karbe H. Koch I. Weiss P. H. (2011). Apraxia impairs intentional retrieval of incidentally acquired motor knowledge. *J. Neurosci.* 31 8102–8108. 10.1523/jneurosci.6585-10.2011 21632932 PMC6622879

[B11] Dragovic M. (2004). Categorization and validation of handedness using latent class analysis. *Acta. Neuropsychiatr.* 16 212–218. 10.1111/j.0924-2708.2004.00087.x 26984309

[B12] Eickhoff S. B. Stephan K. E. Mohlberg H. Grefkes C. Fink G. R. Amunts K. (2005). A new SPM toolbox for combining probabilistic cytoarchitectonic maps and functional imaging data. *Neuroimage* 25 1325–1335. 10.1016/j.neuroimage.2004.12.034 15850749

[B13] Fleminger J. J. Dalton R. Standage K. F. (1977). Age as a factor in the handedness of adults. *Neuropsychologia* 15 471–473. 10.1016/0028-3932(77)90101-4 854167

[B14] Gardini S. Cloninger R. C. Venneri A. (2009). Individual differences in personality traits reflect structural variance in specific brain regions. *Brain Res. Bull.* 79 265–270. 10.1016/j.brainresbull.2009.03.005 19480986

[B15] Gray J. (1987). *The Psychology of Fear and Stress The psychology of fear and stress* 2nd Edn. New York, NY: Cambridge University Press.

[B16] Gray J. McNaughton N. (2003). *The Neuropsychology of Anxiety.* Oxford: Oxford University Press, 444.

[B17] Haber S. N. Knutson B. (2009). The reward circuit: linking primate anatomy and human imaging. *Neuropsychopharmacol* 35 4–26. 10.1038/npp.2009.129 19812543 PMC3055449

[B18] Haider H. Frensch P. A. (2009). Conflicts between expected and actually performed behavior lead to verbal report of incidentally acquired sequential knowledge. *Psychol. Res. Prpf.* 73 817–834. 10.1007/s00426-008- 19034498

[B19] Howard D. Howard J. (1989). Age differences in learning serial patterns: direct versus indirect measures. *Psychol. Aging* 4 357–364. 10.1037//0882-7974.4.3.357 2803630

[B20] Howard D. Howard J. (1992). Adult age differences in the rate of learning serial patterns: evidence from direct and indirect tests. *Psychol. Aging* 7 232–241. 10.1037/0882-7974.7.2.232 1610513

[B21] Izuma K. Saito D. N. Sadato N. (2008). Processing of social and monetary rewards in the human striatum. *Neuron* 58 284–294. 10.1016/j.neuron.2008.03.020 18439412

[B22] Kaasinen V. Maguire R. Kurki T. Brück A. Rinne J. (2005). Mapping brain structure and personality in late adulthood. *Neuroimage* 24 315–322. 10.1016/j.neuroimage.2004.09.017 15627574

[B23] Kennedy K. M. Raz N. (2005). Age, sex and regional brain volumes predict perceptual-motor skill acquisition. *Cortex* 41 560–569. 10.1016/s0010-9452(08)70196-5 16042032

[B24] King B. R. Fogel S. M. Albouy G. Doyon J. (2013). Neural correlates of the age-related changes in motor sequence learning and motor adaptation in older adults. *Front. Hum. Neurosci.* 7:142. 10.3389/fnhum.2013.00142 23616757 PMC3628357

[B25] Lebreton M. Barnes A. Miettunen J. Peltonen L. Ridler K. Veijola J. (2009). The brain structural disposition to social interaction. *Eur. J. Neurosci.* 29 2247–2252. 10.1111/j.1460-9568.2009.06782.x 19490022

[B26] Lin A. Adolphs R. Rangel A. (2012). Social and monetary reward learning engage overlapping neural substrates. *Soc. Cogn. Affect. Neur.* 7 274–281. 10.1093/scan/nsr006 21427193 PMC3304477

[B27] Linke J. Kirsch P. King A. V. Gass A. Hennerici M. G. Bongers A. (2010). Motivational orientation modulates the neural response to reward. *Neuroimage* 49 2618–2625. 10.1016/j.neuroimage.2009.09.013 19770058

[B28] Martin L. E. Potts G. F. (2004). Reward sensitivity in impulsivity. *Neuroreport* 15:1519. 10.1097/01.wnr.0000132920.12990.b9 15194887

[B29] Meier B. Cock J. (2014). Offline consolidation in implicit sequence learning. *Cortex* 57 156–166. 10.1016/j.cortex.2014.03.009 24861420

[B30] Nemeth D. Janacsek K. (2011). The dynamics of implicit skill consolidation in young and elderly adults. *J. Gerontol. Ser. B* 66 15–22. 10.1093/geronb/gbq063 20929973

[B31] Neto L. Oliveira E. Correia F. Ferreira A. (2008). The human nucleus accumbens: where is it? a stereotactic, anatomical and magnetic resonance imaging study. *Neuromodulation* 11 13–22. 10.1111/j.1525-1403.2007.00138.x 22150987

[B32] Nikooyan A. A. Ahmed A. A. (2015). Reward feedback accelerates motor learning. *J. Neurophysiol.* 113 633–646. 10.1152/jn.00032.2014 25355957

[B33] Nissen M. Bullemer P. (1987). Attentional requirements of learning: evidence from performance measures. *Cogn. Psychol.* 19 1–32. 10.1016/0010-0285(87)90002-8

[B34] O’Doherty J. Kringelbach M. Rolls E. Hornak J. Andrews C. (2001). Abstract reward and punishment representations in the human orbitofrontal cortex. *Nat. Neurosci.* 4 95–102. 10.1038/82959 11135651

[B35] O’Doherty J. P. (2004). Reward representations and reward-related learning in the human brain: insights from neuroimaging. *Curr. Opin. Neurobiol.* 14 769–776. 10.1016/j.conb.2004.10.016 15582382

[B36] Oldfield R. (1971). The assessment and analysis of handedness: the Edinburgh inventory. *Neuropsychologia* 9 97–113. 10.1016/0028-3932(71)90067-4 5146491

[B37] Palminteri S. Lebreton M. Worbe Y. Hartmann A. Lehéricy S. Vidailhet M. (2011). Dopamine-dependent reinforcement of motor skill learning: evidence from gilles de la tourette syndrome. *Brain* 134 2287–2301. 10.1093/brain/awr147 21727098

[B38] Parvaz M. A. Konova A. B. Tomasi D. Volkow N. D. Goldstein R. Z. (2012). Structural Integrity of the Prefrontal Cortex Modulates Electrocortical Sensitivity to Reward. *J. Cogn. Neurosci.* 24 1560–1570. 10.1162/jocn_a_00166 22098260 PMC4353578

[B39] Pollock A. Baer G. Campbell P. Choo P. Forster A. Morris J. (2014). Physical rehabilitation approaches for the recovery of function and mobility following stroke. *Cochrane Database Syst. Rev.* 4:CD001920. 10.1002/14651858.cd001920.pub3 24756870 PMC6465059

[B40] Preuss U. W. Rujescu D. Giegling I. Watzke S. Koller G. Zetzsche T. (2008). Psychometrische evaluation der deutschsprachigen version der barratt-Impulsiveness-Skala. *Der Nervenarzt* 79 305–319. 10.1007/s00115-007-2360-7 18210044

[B41] Quattrocchi G. Greenwood R. Rothwell J. C. Galea J. M. Bestmann S. (2017). Reward and punishment enhance motor adaptation in stroke. *J. Neurol. Neurosurg. Psychiatr.* 88 730–736. 10.1136/jnnp-2016-314728 28377451

[B42] Rademacher L. Salama A. Gruender G. Spreckelmeyer K. N. (2014). Differential patterns of nucleus accumbens activation during anticipation of monetary and social reward in young and older adults. *Soc. Cogn. Affect. Neur.* 9 825–831. 10.1093/scan/nst047 23547243 PMC4040093

[B43] Raz N. Rodrigue K. M. (2006). Differential aging of the brain: patterns, cognitive correlates and modifiers. *Neurosci. Biobehav. Rev.* 30 730–748. 10.1016/j.neubiorev.2006.07.001 16919333 PMC6601348

[B44] Reynolds J. Wickens J. R. (2002). Dopamine-dependent plasticity of corticostriatal synapses. *Neural Networks* 15 507–521. 10.1016/s0893-6080(02)00045-x 12371508

[B45] Robertson E. M. (2007). The serial reaction time task: implicit motor skill learning? *J. Neurosci.* 27 10073–10075. 10.1523/jneurosci.2747-07.2007 17881512 PMC6672677

[B46] Robertson E. M. (2009). From creation to consolidation: a novel framework for memory processing. *Plos Biol.* 7:e1000019. 10.1371/journal.pbio.1000019 19175290 PMC2631067

[B47] Robertson E. M. Pascual-Leone A. Miall C. R. (2004). Current concepts in procedural consolidation. *Nat. Rev. Neurosci.* 5 576–582. 10.1038/nrn1426 15208699

[B48] Schuerbeek P. Baeken C. Raedt R. Mey J. Luypaert R. (2011). Individual differences in local gray and white matter volumes reflect differences in temperament and character: a voxel-based morphometry study in healthy young females. *Brain Res.* 1371 32–42. 10.1016/j.brainres.2010.11.073 21126511

[B49] Simon J. J. Walther S. Fiebach C. J. Friederich H.-C. Stippich C. Weisbrod M. (2010). Neural reward processing is modulated by approach- and avoidance-related personality traits. *Neuroimage* 49 1868–1874. 10.1016/j.neuroimage.2009.09.016 19770056

[B50] Steel A. Silson E. H. Stagg C. J. Baker C. I. (2016). The impact of reward and punishment on skill learning depends on task demands. *Sci. Rep.* 6 1–9. 10.1038/srep36056 27786302 PMC5081526

[B51] Struijs J. N. van Genugten M. Evers S. Ament A. Baan C. A. van den Bos G. (2005). Modeling the future burden of stroke in the netherlands. *Stroke* 36 1648–1655. 10.1161/01.str.0000173221.37568.d2 16002757

[B52] Sugawara S. K. Tanaka S. Okazaki S. Watanabe K. Sadato N. (2012). Social rewards enhance offline improvements in motor skill. *Plos One* 7:e48174. 10.1371/journal.pone.0048174 23144855 PMC3492334

[B53] Wächter T. Lungu O. V. Liu T. Willingham D. T. Ashe J. (2009). Differential effect of reward and punishment on procedural learning. *J. Neurosci.* 29 436–443. 10.1523/jneurosci.4132-08.2009 19144843 PMC2765863

[B54] Wickens J. R. Reynolds J. N. Hyland B. I. (2003). Neural mechanisms of reward-related motor learning. *Curr. Opin. Neurobiol.* 13 685–690. 10.1016/j.conb.2003.10.013 14662369

[B55] Yordanova J. Kirov R. Kolev V. (2015). Increased performance variability as a marker of implicit/explicit interactions in knowledge awareness. *Front. Psychol.* 6:1957. 10.3389/fpsyg.2015.01957 26779047 PMC4688353

[B56] Zink C. F. Pagnoni G. Martin-Skurski M. E. Chappelow J. C. Berns G. S. (2004). Human striatal responses to monetary reward depend on saliency. *Neuron* 42 509–517. 10.1016/s0896-6273(04)00183-7 15134646

