# Supplementary figures and images for: Differential Impact of Social and Monetary Reward on Procedural Learning and Consolidation in Aging and Its Structural Correlates
Source: Front Aging Neurosci. 2019 Jul 30;11:188. doi: 10.3389/fnagi.2019.00188 (PMC6682642; doi:10.3389/fnagi.2019.00188)

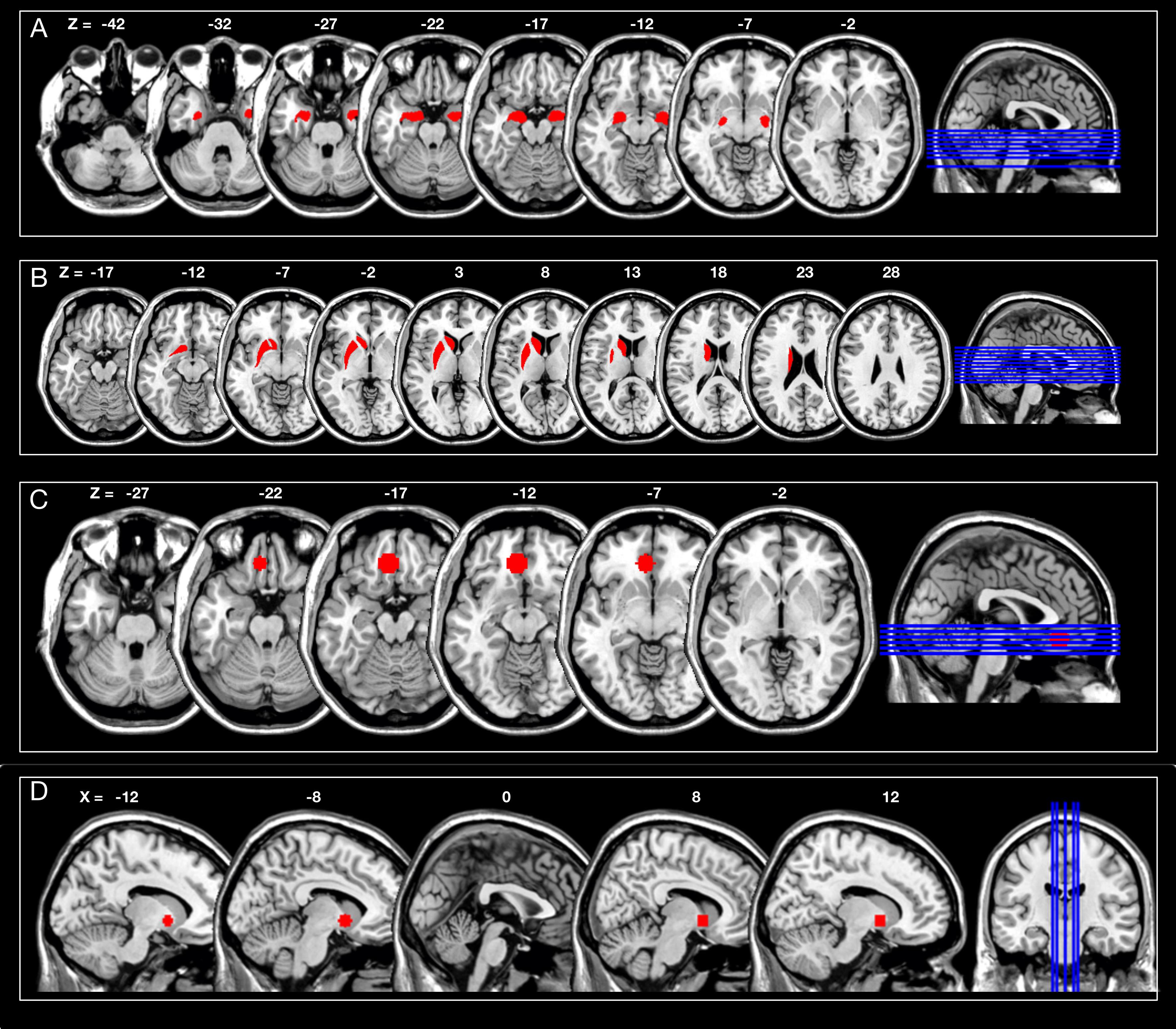

Supplement: FIGURE S1 — Masks for the region of interest (ROI) analysis of voxel-based morphometry (VBM) data. (A) Amygdala [provided by the SPM anatomy toolbox (Eickhoff et al., 2005)]. (B) left Striatum [generated manually with MRIcron (Version 6)]. (C) medial orbitofrontal cortex (mOFC) [defined according to functional imaging data (Lin et al., 2012)]. (D) Nucleus accumbens (NAcc) [defined according to a stereotactic investigation (Neto et al., 2008)]. The masks are displayed on axial (A–C) and sagittal (D) slices of the standard brain provided by MRIcron. x- and z-coordinates refer to the MNI-space. [file Image_1.JPEG]

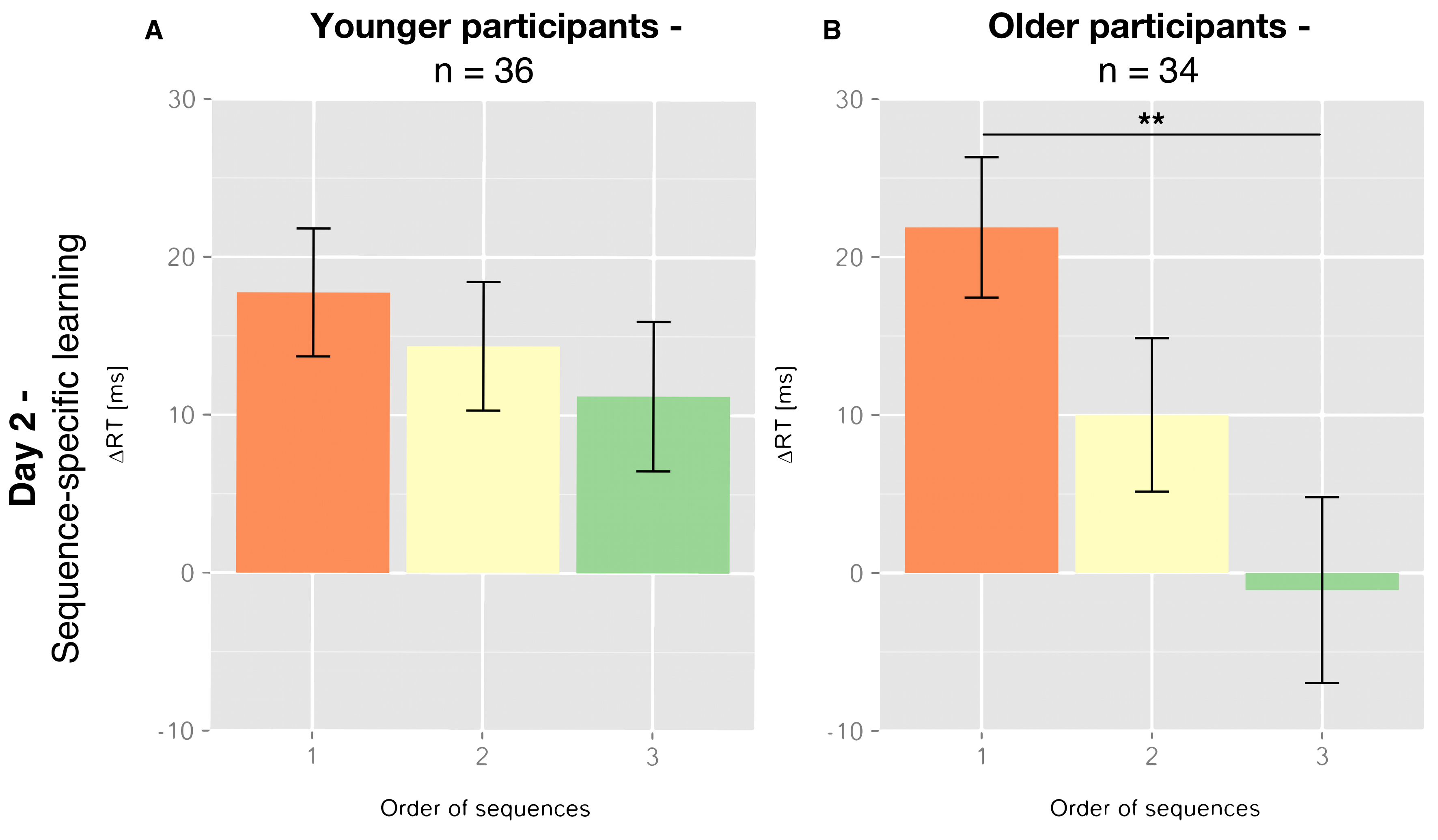

Supplement: FIGURE S2 — Sequence-specific learning (SSL) effects of both groups on day 2 depending on the order of sequences learnt on day 1. Sequence-specific learning (SSL) effects for day 2 are calculated by subtracting the median reaction time (RT) of the respective sequence block from the mean of the median reaction times (RT) of all random blocks (r1, r3, r5, r7). SSL effects on day 2 are depicted for young (A) and older (B) participants depending on the order in which the sequences had been learnt on day 1. Depicted are the means ± standard error of the mean (∗∗p < 0.01). [file Image_2.JPEG]
